# Specific Motor and Cognitive Performances Predict Falls during Ward-Based Geriatric Rehabilitation in Patients with Dementia

**DOI:** 10.3390/s20185385

**Published:** 2020-09-20

**Authors:** Klaus Hauer, Ilona Dutzi, Katharina Gordt, Michael Schwenk

**Affiliations:** 1 Department of Geriatric Research, AGAPLESION Bethanien-Hospital/Geriatric Centre at the Heidelberg University, 69126 Heidelberg, Germany; khauer@bethanien-heidelberg.de (K.H.); idutzi@bethanien-heidelberg.de (I.D.); 2 Institute of Sports and Sports Sciences, Heidelberg University, 69120 Heidelberg, Germany; katharina.gordt@issw.uni-heidelberg.de; 3 Network Aging Research (NAR), Heidelberg University, 69115 Heidelberg, Germany

**Keywords:** fall prediction, dementia, hospitalization, neuro-psychological assessment, instrumented motor assessment, discriminative validity, predictive validity

## Abstract

The aim of this study was to identify in-hospital fall risk factors specific for multimorbid hospitalized geriatric patients with dementia (PwD) during hospitalization. Geriatric inpatients during ward-based rehabilitation (*n* = 102; 79.4% females; 82.82 (6.19) years of age; 20.26 (5.53) days of stay) were included in a comprehensive fall risk assessment combining established clinical measures, comprehensive cognitive testing including detailed cognitive sub-performances, and various instrumented motor capacity measures as well as prospective fall registration. A combination of unpaired t-tests, Mann–Whitney-U tests, and Chi-square tests between patients with (“in-hospital fallers”) and without an in-hospital fall (“in-hospital non-fallers”), univariate and multivariate regression analysis were used to explore the best set of independent correlates and to evaluate their predictive power. In-hospital fallers (*n* = 19; 18.63%) showed significantly lower verbal fluency and higher postural sway (*p* < 0.01 to 0.05). While established clinical measures failed in discriminative as well as predictive validity, specific cognitive sub-performances (verbal fluency, constructional praxis, *p* = 0.01 to 0.05) as well as specific instrumented balance parameters (sway area, sway path, and medio-lateral displacement, *p* < 0.01 to 0.03) significantly discriminated between fallers and non-fallers. Medio-lateral displacement and visuospatial ability were identified in multivariate regression as predictors of in-hospital falls and an index combining both variables yielded an accuracy of 85.1% for fall prediction. Results suggest that specific cognitive sub-performances and instrumented balance parameters show good discriminative validity and were specifically sensitive to predict falls during hospitalization in a multimorbid patient group with dementia and an overall high risk of falling. A sensitive clinical fall risk assessment strategy developed for this specific target group should include an index of selected balance parameters and specific variables of cognitive sub-performances.

## 1. Introduction

Among older persons at risk of falling, persons with dementia (PwD) or cognitive impairment (CI) represent the highest risk populations with a twice as high fall risk compared to cognitively unimpaired, and a fall incidence of 70–85% per year [1,2]. In institutionalized settings, fall rates are dramatic, especially so in PwD/CI, with a threefold fall risk compared to cognitively unimpaired patients [3]. During a considerably shorter observation period as compared to annual fall rates as the established incidence rate, 17% of geriatric rehabilitation hospital patients in mixed populations with respect to cognitive status sustain a fall [4], while about 40% of patients with dementia in psychogeriatric wards fall at least once during a hospital stay [5]. 

Falls occur as an interaction of multiple individuum-related and environment-related risk factors [2,6], with fall risk drastically increasing as the number of risk factors, such as motor impairment, polypharmacy, history of falls, advancing age, female gender, and visual impairment, increases [6,7]. The elevated fall risk in PwD relates to disease-specific impairments of motor function, such as impaired gait, balance, and reduced functional mobility [1,2,8], as well as deficits in specific cognitive subdomains consistently related to increased fall risk, such as impaired executive function [9]. Behavioral disturbances (e.g., wandering) along with psychotropic medication [1,2,10] and lack of self-awareness for functional and cognitive impairments further contribute to fall risk in PwD [11,12].

In this vulnerable patient group with dementia, fall risk is additionally increased by the hospital setting representing an environmental risk factor for falls. The interaction of hospitalization-specific factors, such as unfamiliar physical surrounding and need of assistance [13], with behavioral and psychiatric symptoms affecting judgment and ability to safely negotiate in the environment negatively contribute to fall risk [14]. Moreover, hospitalized PwD are likely to experience progression of behavioral and psychological symptoms due to hospitalization-related distress and disruption of routine [15] and to develop further geriatric syndromes, such as delirium, functional decline, and hospital-acquired incontinence [16], further increasing their risk of falls. 

Falls may represent critical landmarks in older adults’ lives leading to a wide range of adverse events, such as increased morbidity, deconditioning and delayed recovery, mobility decline, post fall anxiety, reduced quality of life, nursing home admission, and increased mortality [17,18]. For PwD, consequences of falls are even more pronounced. Compared to peers without CI, PwD have a substantially increased risk of serious injuries, such as a twofold higher rate of hip fracture [1,19], as well as decreased functional recovery [20]. In hospitalized PwD, falls result in a longer length of stay (LOS) in hospitals and up to a 4.5 times higher mortality rate following hospitalization compared to those without dementia [19], with the mortality risk soaring up tenfold with at least one adverse clinical event, such as a fall, during hospitalization [21].

In this respect, hospitalized persons with dementia represent a particularly vulnerable population with an urgent need for adequate assessment to identify persons with the highest fall risk. To date, various approaches have been used to predict falls in various settings, including clinical assessments with a dual use for clinical routines, such as the geriatric assessment or use as variables for fall prediction [22,23]. These clinical assessments include multiple domains, such as functional, psychological, cognitive, or social status, using different assessment strategies, such as anamnesis (e.g., subjective report on previous falls, use of assistive devices), standardized assessments, using reporting by validated questionnaires or test-based measures (e.g., Falls Efficacy Scale-International (FES-I), Mini-Mental State Examination (MMSE)) and also including established capacity measures, (e.g., Short Physical Performance Battery (SPPB), Timed Up Go (TUG)) [24]. 

However, with most of the assessments being developed for various screening purposes and for non-institutionalized older people, or persons without CI, they may not accurately predict falls in hospital settings [13,25], or may not be applicable in PwD [26], with reduced ability to follow instructions and difficulties to keep up attention or motivation, resulting in potential inappropriateness of established assessment tools or instruments to assess fall risk. 

Previous studies identified single cognitive sub-performances as potential risk factors for falls in community-dwelling older persons [9], but no comprehensive approach including a wider range of cognitive subdomains have been undertaken in PwD.

In systematic reviews on specific fall risk assessments in institutionalized settings, which were not specified for cognitive status despite the high incidence of cognitive impairment in hospital settings, none of the identified established fall risk assessments presented with sufficient accuracy to identify persons with the highest risk for falls [27,28,29]. Among those clinical fall risk assessments, even the one which received a superior rating (St. Thomas’s Risk Assessment Tool (STRATIFY) [30] presented with limitations due to inhomogeneous results depending on the setting and population [28] and a general low predictive accuracy [27,31]. 

Two systematic reviews with a focus on fall risk assessments for PwD documented the inferiority of established clinical fall risk assessment, such as the Performance Oriented Mobility Assessment (POMA) and TUG, compared to objective instrumented measures for gait analysis [22,23]. 

The combination of risk factors from different domains, such as mobility measures, with clinical fall risk assessment tools documenting cognition, health status, or age improved the accuracy of fall prediction in PwD in non-hospital settings [32,33,34]. While the combination with global measures of cognition had no effect on the accuracy of fall prediction [33,34], specific cognitive measures, such as executive function, increased the accuracy for multiple falls in community-dwelling older persons with mild to moderate CI [34]. 

In summary, there is evidence suggesting an added value for combined fall prediction models in community-dwelling PwD. However, no study has evaluated such combined models in hospitalized PwD. It is not clear which clinical, motor, and cognitive factors are useful for developing accurate fall prediction assessment in this specific setting. A PwD-tailored assessment approach combining the most relevant variables derived from clinical assessment, motor assessment (i.e., instrumented gait and balance analysis), and comprehensive cognitive assessment including multiple subdomains could be a useful approach to identify fall-prone PwD in a hospital setting.

It therefore was the objective of this study to identify risk factors for falls combining different domains with high association to the target group of older multimorbid hospitalized persons with a confirmed dementia diagnosis.

## 2. Materials and Methods

### 2.1. Study Design and Method

We present a cohort study of geriatric PwD consecutively recruited during ward-based rehabilitation. Data collection started within 48 h after admission and included predominantly baseline measures with the exception of the number/incidence of falls, which was documented at the end of rehabilitation based on strictly standardized electronic patient charts. The study was part of the project “Geriatric Rehabilitation for Patients with Dementia Study” (GREDE) conducted at the AGAPLESION Bethanien Hospital/Geriatric Center, Heidelberg University, Germany [35] and was approved by the Ethics Committee of the Medical Faculty at Heidelberg University in accordance with the Helsinki Declaration.

The inclusion criterion for GREDE was the diagnosis of mild to moderate dementia as a secondary diagnosis confirmed by a geriatrician according to core criteria for all-cause dementia based on a standardized approach, including clinical history, physical and neurological examination, neuroimaging, laboratory tests, and neuropsychological testing [36]. Exclusion criteria were medical and/or psychological conditions not allowing the application of neuropsychological and functional assessments, such as acute confusion, aphasia, severe visual or auditory impairment, severe psychiatric disorders, severe functional-motor deficits, inadequate language level, or lack of written informed consent by participants or their legal representatives. 

Each participant received personalized rehabilitation, depending on individual abilities and rehabilitation needs based on comprehensive geriatric assessments. Interventions included exercise, physiotherapy, assistive technology, psychological, and social interventions. The coordinated multidisciplinary team of health professionals involves physicians, nurses, physical and occupational therapists, speech language pathologists, psychologists, therapeutic recreation therapists, as well as social workers. A main focus of the routine geriatric rehabilitations is given to established rehab goals, such as functional strength (e.g., as documented by sit-to-stand performances), stair climbing, walking performances, or postural control as basic key motor features to regain or defend autonomy of this vulnerable study population.

### 2.2. Measurements

Measures were assessed by trained research assistants and comprised well-established assessments, validated in older persons and in cognitively impaired patients.

#### 2.2.1. Patient Characteristics

Patient characteristics included age, gender, global cognitive status (MMSE, range 0–30) [37], activities of daily living (Barthel-Index, range 0–100) [38,39], indication for geriatric rehabilitation by diagnostic groups as documented in patient charts, number of medications, depressive symptoms (Geriatric Depression Scale (GDS), 15-item version) [40], frailty status (Clinical Frailty Scale (CFS), range 1–9) [41], physical activity during ward-based rehabilitation (Physical Activity of Inpatient Rehabilitation questionnaire (PAIR), range 0–7) [42], and length of stay (days).

#### 2.2.2. Definition of Outcomes

- Documentation of in-hospital falls

Outcomes were “in-hospital fallers” versus “non-fallers”. “In-hospital fallers” were defined as patients having one or more falls during ward-based rehabilitation. Classification was based on documentation in hospital charts as part of the hospital critical incident reporting system and risk management during the 3-week rehabilitation period using the standard definition of a fall as “an incident in which a patient suddenly and involuntarily comes to rest upon the ground or a surface lower than their original situation” [43].

- Fall risk assessed by fall risk screening instrument

Risk for in-hospital falls was assessed by the STRATIFY risk assessment [30]. The well-established instrument has been developed to predict patients at high risk of falling [28]. It comprises five questions about the absence (score 0) or presence (score 1) of fall risk factors: (1) previous falls, (2) disorientation, mental agitation (3) visual impairment, (4) frequent toileting, and (5) mobility impairment (score 15–20 in the Barthel Index mobility subscale). Thus, the score ranges from 0 (low falls risk) to 5 (high falls risk), with a score of 2 or more indicating high fall risk [30].

- Fall-associated clinical measures

Based on the literature review, we identified fall-associated characteristics, including age, sex, number of medication, previous fallers (defined as people who had at least one injurious fall or at least two non-injurious falls during the last 12 months [44,45]), concerns about falling (measured with the Short Falls Efficacy Scale-International (Short FES-I; range 7–28) [46]), global cognitive functioning (MMSE), and motor capacity measures [8,22,47,48,49].

- Fall-associated motor capacity measures

Muscle strength was documented by the standardized one repetition maximum (1-RM) achieved at a leg-press training machine for maximal dynamic concentric strength in hip and knee extensors (in kg) (Kaphingst, Lahntal, Germany) and the 5-chair stand test as an established functional assessment in older adults [50]. The Performance Oriented Mobility Assessment (POMA) assessed a person’s mobility and requires both static and dynamic balance abilities [51] (maximal score 28). The Timed Up and Go test (TUG) is a reliable and valid clinical test to quantify functional mobility. The time needed, in seconds, to stand up from a regular arm chair, walk a 3-m distance at a comfortable pace, turn around, return to the chair, and sit-down again is measured [52,53].

- Assessment of cognitive subdomains

Cognitive subdomains commonly affected in demented patients were comprehensively assessed by subtests of the Consortium to Establish a Registry for Alzheimer’s Disease Neuropsychological Assessment Battery (CERAD) [54], including aspects of five different cognitive domains: executive functioning (verbal fluency: number of words starting with the letter S generated in 60 s), language semantic memory (modified version of the Boston Naming Test: naming 15 objects presented as line drawings, maximum score (MS) = 15); episodic memory encoding and recall (Word List-Learning): sum of words learned in three trials in 10-word learning list, MS = 30 and Word List –delayed recall: delayed recall of the 10 words presented in WL, MS = 10); visuospatial abilities (Constructional Praxis: figures—copy, MS = 11); and speed of information processing (Modified Trail Making Test from the Nuremberg Gerontopsychological Inventory [55]: connecting numbers, mean time of 2 trials, max. 300 s).

- Instrumented measures for gait and balance

Temporo-spatial gait capacity (gait speed, cadence, and stride length) was obtained using the pressure-sensitive GAITRite-system (CIRSystems, Havertown, PA, USA; length: 4.8 m). Subjects walked with maximum speed using a walking aid if necessary [56].

Balance capacity was assessed by a wearable sensor (DynaPort^®^ Hybrid, McRoberts, The Hague, The Netherlands) consisting of a tri-axial accelerometer inserted in an elastic belt and positioned on the patients’ lower back at the height of the second lumbar vertebra [57]. Balance capacity was assessed during quiet standing with feet close together (parallel stand) for a 30-s period while data analysis was executed automatically using online software (http://www.mcroberts.nl/analysis). Center of mass sway area (mm^2^), sway path (mm/s), and the root mean square (RMS) of anterior-posterior and medio-lateral displacement (mm) were documented [57]. The RMS indicates the average amplitude of sway in the respective direction [58].

During the five-chair stand tests, the patients also wore the wearable sensor DynaPort^®^ Hybrid. They were instructed to come to a full upright standing position and back to the initial sitting position in between each sit-to-stand cycle. A sit-to-stand cycle is comprised of sitting, standing up, standing, sitting down and sitting. After an automated identification of these phases, the duration of the sit-to-stand and stand-to-sit phase and the duration of the flexion and extension phase of the sit-to-stand and stand-to-sit phase (in seconds) were calculated [59].

### 2.3. Statistical Analysis

Socio-demographic and clinical characteristics of participants were documented as frequencies (n, %), means with standard deviations, or medians with interquartile ranges as appropriate for the distribution of the variables. 

#### 2.3.1. Discrimination between In-Hospital Fallers and Non-Fallers

Unpaired t-tests, Mann–Whitney-U tests, and Chi-square tests as appropriate were used for comparison of patient characteristics between patients with (“in-hospital fallers”) and without an in-hospital fall (“in-hospital non-fallers”). As sample sizes were unequal between groups, we used Welch’s t-test as the default strategy. 

#### 2.3.2. Prediction of In-Hospital Falls

A two-step approach was applied to identify the best set of independent predictors with in-hospital-falls in the study sample. In a first step, we used univariate analysis to describe transparently the associations between the dichotomized criterion “in-hospital faller/non-faller” and fall-associated variables found statistically different between subgroups [60]. In the second step, variables significantly correlated in the univariate analysis (*p* ≤ 0.05) were subsequently included in in the final multivariate forward stepwise analysis to explore the best set of independent correlates and to evaluate their predictive power. Effects were quantified by odds ratios (ORs) with corresponding 95% confidence intervals (95% CIs) and coefficient of determination (R^2^). All variables were treated as continuous except “previous faller”, which was treated dichotomously (yes/no). 

For the variables remaining significantly in the multivariate analysis, the receiver operating curve (ROC), the area under the curve (AUC), and the accuracy (%) were calculated in three different fall-prediction models (single versus combined models). 

A two-sided *p*-value ≤ 0.05 was considered to be statistically significant. Statistical analyses were performed using SPSS statistics 25.0 (IBM, Armonk, NY, USA).

## 3. Results

The sample population comprised 102 multimorbid (number of medication: 9.9 ± 3.3), high-aged inpatients (82.82 ± 6.19 years of age) with cognitive (MMSE score: median: 22; IQR: 20.75–24) and functional impairment (Barthel Index: median: 65; IQR: 55–80) (see Table 1). 

### 3.1. Discriminating between In-Hospital Fallers and Non-Fallers

During the study period, 19 patients (18.63%; 84.2% females) sustained an in-hospital fall. Table 2 presents the characteristics and differences between the two subgroups (in hospital fallers vs. non-fallers). The established clinical fall risk assessment instrument STRATIFY indicated a high risk of falling in both groups (median = 2) but did not discriminate between groups (*p* = 0.22). Comparisons between groups indicated no differences for global cognitive functioning, age, gender, number of medications, previous falls, and established motor capacity measures (POMA, TUG, 5-chair rise, and 1RM) (*p* = 0.10 to 0.97). 

In contrast, significant differences were found in measures of the comprehensive cognitive assessments and instrumented measures for gait and balance. While the Boston naming task, the Trail-Making Test, and the memory tasks did not discriminate between subgroups, in-hospital fallers showed significantly lower scores in the verbal fluency (mean z-score = −1.76 (SD 0.93) vs. mean z-score = −2.26 (SD 1.01) and the constructional praxis task (mean z-score = −1.93 (SD 1.20) vs. mean z-score = −2.78 (SD 1.51)) compared to the non-fallers indicating better performance in the cognitive subdomains of executive (*p* = 0.039) and visuospatial abilities (*p* = 0.01) in patients who did not fall during hospital stay. 

Additionally, significant differences were found for the instrumented measures sway area (*p* = 0.05) and sway path (*p* < 0.01) as well as highly significant differences for medio-lateral displacement (*p* < 0.01), indicating better static balance ability in patients who did not fall during hospital stay. Instrumented gait and sit-to-stand measures did not differ between groups.

### 3.2. Prediction of In-Hospital Falls

Two cognitive subdomain measures, verbal fluency (*p* = 0.05) and constructional praxis (*p* = 0.01), and the three sensor-based balance measures from the sway analysis (medio-lateral displacement, sway path, and sway area; *p* < 0.01 to 0.03) were significantly associated with in-hospital falls according to univariate regression analysis (Table 3). Medio-lateral displacement explained the highest proportion of variance (Nagelkerkes R^2^ = 0.18) (Table 3). 

Based on the results of the univariate logistic regression analyses, significant variables were included in the multivariate logistic regression analysis to explore the best set of independent correlates with in-hospital falls. 

In the multivariate analysis, only the two variables “constructional praxis” (*p* = 0.05) and “medio-lateral displacement” (*p* = 0.01) remained as significant predictors for in-hospital falls, explaining 24% of the variance (Table 4). Lower performance in visuospatial abilities and higher medio-lateral displacement during standing was associated with a higher risk for in-hospital falls (OR: 0.57, *p* = 0.01 and OR: 1.25, *p* < 0.01, respectively) in our sample. 

For the significant variables that were identified in the multivariate analysis, the receiver operating curve (ROC), the area under the curve (AUC), and the accuracy (%) were calculated in three different fall-prediction models (univariate versus combined models).

**Figure 1:**
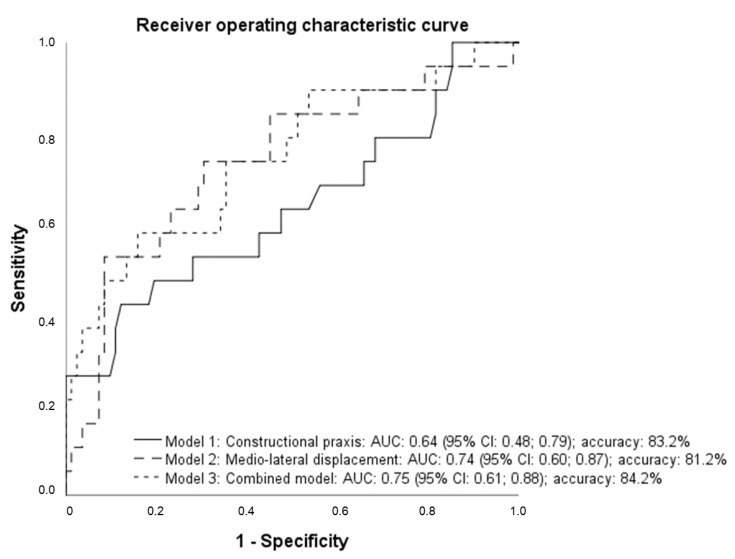
Receiver operating characteristic curve for the two individual and the combined predictors for in-hospital falls in patients with dementia. Presented are receiver operating curves of different models for predicting future in-hospital falls in PwD: Model 1 used ‘constructional praxis’ (AUC: 0.64), model 2 used ‘medio-lateral displacement’ (AUC: 0.74), and model 3 combined these two variables to a combined model (0.75).

Predictive properties were determined using receiver operating characteristic (ROC) curves with in-hospital falls as the criterion variable and “constructional praxis” (model 1), “medio-lateral displacement” (model 2), and a combination of both variables (model 3) as the independent variables. The ROCs are shown in Figure 1. “Constructional praxis” (model 1) had an AUC of 0.62 (95% CI: 0.45–0.79) and an accuracy of 83.2% for predicting in hospital falls. “Medio-lateral displacement” (model 2) had an AUC of 0.74 (95% CI: 0.60–0.87) and an accuracy of 81.2%. The index combining these variables (model 3) showed the highest AUC (0.75; 95% CI: 0.61–0.88) and most predictive accuracy (84.2%) (Figure 1). 

## 4. Discussion

This study evaluated the discriminative and predictive validity of parameters to identify in-hospital falls in multimorbid people with confirmed mild to moderate stage dementia. To our knowledge, this is the first study using a combined methods approach, which compared clinically established measures, neuropsychological variables for detailed cognitive sub-performances, and instrumented objective motor capacity parameters to predict in-hospital falls in PwD. Our findings suggest that both motor biomarkers derived from instrumented assessments and specific cognitive variables based on comprehensive neuro-psychological tests are superior to traditional fall risk assessment in this high risk in-hospital population with dementia.

- Clinical measures

In the present study, we included clinically established measures, which have all been identified as fall risk factors in previous studies. These multi-domain variables comprise social, psychological, functional, health status, and demographic variables documented in established clinical routines, such as geriatric assessment. They are therefore available in most patient-related hospital data sets. These variables were included to cover most relevant clinical information in this specific rehab setting with acutely impaired multimorbid persons, in which such information seemed mandatory and highly relevant for the analysis of in-hospital fall risk factors. 

Results of the present study, however, indicate that these measures have only limited relevance for the identification of risk of falling in the studied population. None of the included clinical variables neither allowed a significant discrimination between fallers and non-fallers, nor was identified as a predictor of falls in the univariate or multivariate regression models. Additionally, a well-established fall assessment tool for in-hospital falls (STRATIFY) based on a number of such single clinical measures showed similar negative results. The present results are in line with systematic reviews indicating only a limited predictive validity of such specific risk assessment tools for in-hospital falls [25,26,27,29] or for single clinical variables, such as established functional tests [22,23,61] or other assessments established in clinical settings [24]. 

Those limitations may be based on different reasons. Some of the parameters show only moderate associations to falls and may only be highly relevant in subgroups most affected by the parameter [62].

Other parameters are based on subjective reporting with known limitations of accuracy in PwD [63]. For most established measures, no modified version of the assessment tools in order to accommodate for the attention and memory deficit in PwD are available [30], thus limiting the applicability of such measures in PwD. In general, all these parameters have not primarily been developed to identify risk of falling and may therefore not be specific enough to identify the highest risk sub-populations among multimorbid acutely impaired patients with dementia during hospital stay with an overall high risk of falling [61]. This challenging task may serve as a background for deviating results for the use of such parameters in community-dwelling older persons but also for the negative results of the established risk of falling assessment tool (STRATIFY), rated best in systematic reviews among comparable assessment tools [26], which was also analyzed in the present study. STRATIFY correctly identified a general risk of the total sample but not those with the highest risk within such a high-risk group, documenting sufficient sensitivity, but not limited specificity, as already reported in a previous review [27,29].

- Cognitive measures

Cognitive status has long been a neglected domain for fall risk assessment despite the fact that CI represents a major risk factor for falls with direct negative consequences to interventional programs in which related cognitive training approaches have hardly been included [64]. Surprisingly, and in contrast to the high risk of falling in hospitalized PwD, we could not identify comprehensive assessment tools for risk of falling, which were specifically developed for this most affected population [10]. In order to allow an adjusted risk profile in this study for the target sample with diagnostically confirmed dementia, we included a comprehensive cognitive assessment to document various cognitive sub-performances in addition to an established screening measure of global cognitive status (MMSE).

In the present study, the overall cognitive status or specific subdomains (e.g., memory-related performances) did not predict falls neither in the univariate nor multivariate regression model nor discriminated between fallers and non-fallers. In line with previous studies for different settings in older persons with or without CI [33,34,65,66], results indicate that, at least in this rather homogeneous sample with respect to cognitive status, the general cognitive status was not specific and sensitive enough to predict in-hospital falls in PwD and that cognitive sub-domains have high specificity to qualify for fall prediction.

In contrast, specific measures for cognitive sub-performances added to the prediction of such falls. Decreased frontal executive function as operationalized by the verbal fluency test [67] and constructional praxis for visuo-spatial performances [68,69] stood out, showing significant discriminative validity for fallers vs. non fallers in the present study. While verbal fluency and constructional praxis were also significantly associated with in-hospital falls in univariate regression analysis, only constructional praxis survived in a multivariate model. Visuo-spatial results also improved the fall prediction model, including both cognitive and motor variables, which were identified by multiple regression, yielding a comparatively high accuracy for two single parameters of 84% with an AUC of 0.75. The study results thereby document a superior specificity and sensitivity of specific measures of cognitive sub-performances as compared to global cognitive measures and established clinical measures.

The constructional task is a test of visuospatial function or the representation of objects in a spatial array and impairment in this function may impact the ability to navigate safely between two places by judging distances or avoiding objects. However performance on this test also relies upon the integrity of executive functions (organization and planning), which means that the task can be seen to have executive functioning demands as well [70,71]. Many facets of the umbrella term executive function are relevant for risk of fall, including working memory, planning, shifting, fluency, and inhibition, and are often associated to multiple other fall risk factors, such as female gender, higher consumption of centrally acting medications, and poorer functional capacity [34], including balanced impairment as a major contributor to risk of falling as in the present study. We therefore interpret the constructional praxis finding as a specific cognitive indicator for a complex interaction to multiple accumulating fall risks.

The results are in line with other studies identifying visuospatial abilities [71], executive functions [8], and other cognitive sub-performances, such as processing speed [9,34], as risk factors for falls. However, such cognitive risk factors have, to our knowledge, so far not been identified and specified for in-hospital falls and in PwD.

- Instrumented measures

We included instrumented measures as they give a detailed insight into motor functional deficits with a high incidence of partly specific functional limitations in PwD as compared to cognitively intact peers [1,2,8], representing the highest impact factor for falls for the general population [72].

We used instrumented assessment to capture key gait and balance parameters, previously linked to fall risk in PWD, as described in a recent review paper [30]. In line with this review, we found instrumented balance measures as significant predictors for prospective falls in our sample. More specifically, we identified medio-lateral displacement as an independent fall predictor remaining in the final regression model. Increased center of mass sway in the medio-lateral direction has been repeatedly identified as a predictor for future falls [73,74]. From the general model of balance control, for side-by-side standing, the medio-lateral neuromuscular control is a hip load/unload mechanism while anterior-posterior control is controlled at the ankle [75]. Previous studies in subjects with subjective memory impairment have shown that the medio-lateral neuromuscular control mechanism is affected by cognitive impairment, while the anterio-posterior control mechanism is not affected [76]. This may explain our findings about the sensitivity of medio-lateral displacement for predicting falls in PwD. 

We did not find any differences in gait characteristics between fallers and non-fallers. This contrasts the finding of a recent review reporting gait speed and stride length to differentiate between fallers and non-fallers [30]. However, none of the studies included in this review were conducted in a hospital setting with an overall high-risk vulnerable population. Furthermore, no study was conducted for short-term fall prediction within a geriatric rehabilitation period of 20 days but predicted falls within a period of 2 to 24 months. Therefore, a direct comparison of our study results in our specific setting and population with those of previous studies is not possible. We also observed floor effects for gait analysis in some of our participants, which may have limited the predictive validity of the gait analysis. 

People with dementia show disease-specific sit-to-stand movement disorders, which relate to deficits of integrating cognitive aspects of motor processes into motor action organization [77]. Reduced performance in the 5-chair stand test is linked to fall risk in older persons [78]. Results of our sit-to-stand analysis revealed that both groups (fallers and non-fallers) had substantial impairments in the sit-stand transfer, as indicated by the total time needed. However, no significant between-group differences were found, indicating that the 5-chair stand time has limited discriminative validity for fall risk in geriatric inpatients. In order to explore potential differences in specific sub-phases of the sit-stand transfer, we additionally performed an instrumented analysis of the 5-chair stand, based on an approach published previously by our group [59]. We found that the times needed to execute the trunk flexion and extension during both the sit-to-stand and stand-to-sit did not significantly differ between groups. The 5-chair stand test is a motor-demanding task and 23.5% of our participants were unable to complete this assessment at the beginning of the rehabilitation phase. These floor effects reduced the sample size for this specific assessment and may explain the limited sensitivity of this measure for estimating fall risk in our sample. 

In summary, our results for the instrumented assessment suggest that specific balance parameters predict falls in geriatric rehabilitation inpatients, confirming previous findings [8]. In our sample, these parameters had superior predictive validity as compared to instrumented gait and sit-stand variables. They also outperformed the standard motor assessments, such as the TUG and POMA, which is in line with findings from systematic reviews [30].

- Combined measured

We combined fall risk measures as such combinations best cover the complex nature of fall mechanisms, which are based on multiple domains, including clinical, cognitive, and motor risk factors, in the multimorbid hospitalized acutely impaired high-risk sample for falls with dementia.

Using multiple regression analysis, we aimed to identify those variables independently associated with falls in our sample. Variables in the domains of postural sway (i.e., medio-lateral displacement) and cognitive performance (constructional practice) remained in a multivariate model. In line with our results, previous studies using multivariate analysis have reported postural sway to be significantly and independently associated with falls while controlling for age. Impaired balance is a key risk factor for falls in cognitively intact older people [73], and there is increasing evidence that impaired balance is also an important risk factor for falls in the cognitively impaired [30]. Our study emphasizes the importance of postural balance impairment as a key risk factor for experiencing a fall during the geriatric rehabilitation period. The assessed postural balance performance in a quiet standing position can be considered a surrogate measure of overall sensorimotor performance [79]. Our findings suggest that those PWD admitted to rehab with a low sensorimotor performance had a greater risk to experience a fall.

In our study sample, the combination of specific instrumented postural balance parameters (medio-lateral displacement) and specific cognitive deficits (constructional praxis) was the most accurate for predicting falls. Using a similar approach, Taylor et al. (2014) also identified several risk factors in univariate models (first step) and included relevant factors in a multivariate model (second step). In line with our study, authors found that specific cognitive performances, including visuospatial deficits, were significantly associated with fall rates in univariate models [8]. However, in Taylor’s study, cognitive variables did not remain in the multivariate model, as found in our study. 

Several characteristics of Taylor’s and our study are comparable, including age (mean: 82.2 vs. 82.8 years), level of cognitive impairment (mean MMSE 22.7 vs. 22.0 points), and motor impairment (mean TUG: 21.0 vs. 21.2 s). On the same note, studies differ with respect to sample size (*n* = 174 vs. *n* = 102), gender distribution (women: 56% vs. 80%), and follow-up period for monitoring falls (12 months vs. 20 days), which may explain the results discrepancy between both studies. Furthermore, Taylor et al. (2014) included cognitively impaired but not exclusively those with a confirmed dementia diagnosis, as performed in our study. Specific cognitive variables related to dementia may therefore have played a specific role in fall risk assessment in our study. Moreover, Taylor et al. (2014) observed falls risk in a community-dwelling setting while our study was conducted in a geriatric inpatient rehabilitation setting. Systematic review papers have requested tailored fall risk assessments depending on the setting [48]. One could speculate that the specific cognitive deficits found in our sample increased fall risk specifically in the rehabilitation environment and should therefore be considered when assessing fall risk in this setting. In summary, our study findings suggest that the best predictive accuracy can be achieved when combining specific instrumented balance measures and cognitive measures. 

We see the strength of the present study in the combination of different highly relevant domains, such as clinical measures, in-depth neuropsychological assessment, and multiple instrumented measures, with a strong focus on the target population of hospitalized multimorbid geriatric PwD, which, to our knowledge, have not been analyzed before in this comprehensive density for fall prediction purpose in this setting.

As a generic limitation, we refer to the fact that the generalizability of the results is restricted to the study setting and sample, although the results may translate into other samples to a certain extent. As in previous comparable studies in hospital settings, the number of documented falls is limited by the short individual observation period and the random nature of fall events, which are still rare events even in such high-risk populations, with consequences on the modeling to identify risk factors for in-hospital falls. A study with a larger sample size could verify the validity of the parameters found in the present study. We did not include activity-related and environmental factors, which are important in the etiology of falls and may combine with intrinsic risk factors to increase risk [80]. On the same note, the intrinsic fall risk factors found in our study could be modifiable to potentially inform future prevention strategies for the specific setting and population.

## 5. Conclusions

In conclusion, we found that the combination of selected balance parameters and specific variables of cognitive sub-performances has the potential to provide a clinically meaningful surveillance of PwD at high risk of falling. The sensor-based balance assessment used in the present study can be quickly and safely administered during routine care. In contrast to traditional rater-based falls risk assessment, the sensor-based assessment generates an objective unbiased estimate of fall risk. Established global functional status measures, such as the TUG, seem to have limited sensitivity in the ward rehabilitation setting. Instead, our study shows an added value of a specific balance analysis, extending the current guidelines of fall risk assessment. Systematic reviews have pointed out that different types of settings should probably use different assessment approaches [48], but no specific recommendations exist for PwD in a geriatric ward setting. The present findings may help to design a specific assessment strategy for this setting incorporating sensor-based balance assessment and specific assessment of cognitive sub-performances going beyond the currently used cognitive screening instruments. Our study suggests that an assessment of specific cognitive sub-performances could have an added value for predicting falls in this specific setting. The identified variables should be considered in the future development of fall prediction strategies for PwD. Beyond the identification of specific cognitive risk factors, the results may also serve as a first stepping stone to develop specific intervention programs tailored for this specific target sample. Risk factors as identified in this study may qualify as candidates for such specific training targets for future fall prevention programs.

## Figures and Tables

**Table 1:** Participants’ baseline characteristics of the total study sample (*n* = 102).

Variable	All (*n* = 102)
Age, years, mean (SD)	82.82 (6.19)
Gender, female, %	79.4
MMSE score (0–30), median (IQR)	22 (20.75–24)
Barthel-Index (0–100) median (IQR)	65 (55–80)
Indication for geriatric rehabilitation by diagnostic groups, %	
Orthopedic problem	25.0
Cerebrovascular disease	19.3
Heart disease	15.9
Other internal disease	21.6
Miscellaneous	18.2
Medication, n, mean (SD)	9.93 (3.27)
GDS score (0–15), median (IQR)	4.0 (1.25-5)
CFS score (1–9), median (IQR) (*n* = 101)	6 (5–6)
PAIR score (0–7), median (IQR) (*n* = 87)	6 (4–7)
In-hospital fall, yes, %	18.63
Length of stay (days), median (IQR)	20 (19–21)

Presented are baseline values for the total study sample. MMSE, Mini Mental State examination; GDS, Geriatric Depression Scale, short version; CFS Clinical frailty scale; PAIR, Physical Activity in Inpatient Rehabilitation Assessment; Total range of scores are given in brackets behind the variable with higher scores indicating less impairment in MMSE, Barthel Index and PAIR. For GDS and STRATIFY higher scores indicate higher degree symptoms. Actual n was indicated for variables with incomplete data.

**Table 2:** Characteristics and differences between in-hospital fallers and non-fallers.

Variable ^a^	All (*n* = 102)	In-Hospital Non-Fallers (*n* = 83)	In-Hospital Fallers (*n* = 19)	*p*-Value
**Fall risk screening instrument**				
STRATIFY (0–5), median (IQR)	3 (2–3.5)	3 (2–3) (*n* = 82)	3 (2–4)	0.22 ^2^
**Established fall associated clinical measures**				
Age, years	82.82 (6.19)	83.04 (6.01)	81.89 (6.73)	0.47 ^1^
Gender female, %	79.4	78.3	84.2	0.57 ^3^
Medication, n	8.93 (3.27)	9.02 (3.35)	8.53 (2.98)	0.55 ^1^
Fallers (retrospective) ^4^, yes, %	50.0	49.4	52.6	0.80 ^3^
FES-I score (7–28), median (IQR)	10 (8–12)	10 (8–12)	11 (8–14)	0.24 ^2^
MMSE score (0–30), median (IQR)	22.03 (2.59–24)	23 (19–24)	22 (21–24)	0.88 ^2^
**Fall associated motor capacity measures**				
1 RM, kg	110.21 (60.77)	114.27 (60.27) (*n* = 75)	94.21 (61.68).	0.20 ^1^
5-chair stand, s	16.16 (5.91)	16.33 (6.10) (*n* = 70)	15.30 (4.93) (*n* = 14)	0.66 ^1^
POMA score (0-28)	21.07 (6.76)	21.62 (6.59) (n=81)	18.74 (7.19)	0.10 ^1^
TUG, s	21.15 (12.06)	20.86 (12.61) (*n* = 78)	22.38 (9.52) (*n* = 18)	0.64 ^1^
**Assessment of cognitive subdomains (z-scores)**				
Verbal Fluency	−1.85 (0.96)	−1.76 (0.93)	−2.26 (1.01)	0.04 ^1^
Boston Naming	−1.28 (1.11)	−1.31 (1.13)	−1.15 (1.07)	0.57 ^1^
Word List-Learning	−2.90 (1.24)	−2.83 (1.22)	−3.22 (1.31)	0.22 ^1^
Word List- Delayed Recall	−2.01 (1.13)	−1.93 (1.12)	−2.38 (1.10)	0.11 ^1^
Constructional Praxis	−2.09 (1.30)	−1.93 (1.20) (*n* = 82)	−2.78 (1.51)	0.01 ^1^
Trail Making Test	−1.61 (0.83)	−1.57 (0.84) (*n* = 81)	−1.82 (0.76) (*n* = 15)	0.30 ^1^
**Instrumented measures for gait and balance**				
Sway Analysis (DynaPort)				
Sway area, mm^2^	1011.26 (99.98)	892.67 (749.13)	1529.31 (1280.69)	0.05 ^1^
Sway path, mm/s	20.55 (8.11)	19.44 (7.37)	25.39 (9.58)	<0.01 ^1^
Anterior-posterior displacement, RMS, mm	9.15 (4.72)	8.60 (3.58)	11.58 (7.68)	0.11 ^1^
Medio-lateral displacement, RMS, mm	8.66 (3.84)	8.00 (3.34)	11.49 (4.65)	<0.01 ^1^
Gait Analysis (GaitRite)	*n* = 94 (92.2%)	*n* = 76 (91.6%)	*n* = 18 (94.7%)	
Gait speed, cm/s	79.47 (36.00)	80.12 (37.02)	76.74 (32.12	0.72 ^1^
Cadence, steps/min	105.07 (19.50)	104.43 (19.31)	107.82 (20.60)	0.82 ^1^
Stride length, cm	89.04 (29.36)	98.92 (30.32)	85.32 (25.32)	0.25 ^1^
Sit-to-Stand Transfer Analysis (DynaPort)	*n* = 78 (76.5%)	*n* = 64 (77.1%)	*n* = 14 (73.7%)	
Sit-to-Stand duration, s	1.61 (0.44)	1.60 (0.43)	1.65 (0.51)	0.67 ^1^
Sit-to-Stand flexion duration, s	0.81 (0.20)	0.81 (0.20)	0.79 (0.22)	0.78 ^1^
Sit-to-Stand extension duration, s	0.80 (0.26)	0.78 (0.25)	0.86 (0.30)	0.33 ^1^
Stand-to-Sit duration, s	1.65 (0.47)	1.65 (0.50)	1.60 (0.29)	0.72 ^1^
Stand-to-Sit flexion duration, s	0.84 (0.32)	0.85 (0.35)	0.79 (0.17)	0.51 ^1^
Stand-to-Sit extension duration, s	0.81 (0.22)	0.81 (0.23)	0.82 (0.19)	0.21 ^1^

Summary statistics for the two groups of in-hospital fallers and non-fallers. a = Given are mean (SD) unless otherwise stated. Differences were compared using 1 = Unpaired t-test, 2 = Mann–Whitney-U and 3 = Chi-square test. STRATIFY, St. Thomas’s Risk Assessment Tool in falling elderly inpatients; MMSE, Mini Mental State examination; Short FES-I, Short Falls Efficacy Scale-International; TUG, Timed up and go test; 1 RM, Maximal Strength leg press; POMA, Performance Oriented Mobility Assessment; 4 = fallers were defined as persons with >2 falls or at least 1 injurious fall during last year. Total range of scores are given in brackets behind the variable with higher scores indicating less impairment in MMSE and 1 RM and GaitRite analysis. For POMA, TUG, 5 chair rise, FES-I, STRATIFY and DynaPort analyses higher scores indicate higher degree symptoms. Actual n was indicated for variables with incomplete data.

**Table 3:** Results of the univariate logistic regression and area under the receiver operating characteristic curve (AUC) analysis.

Variable	∆R^2^	OR	95% CI	*p*-Value
**Cognitive subdomains**				
Verbal Fluency (z-scores)	0.07	0.58	0.34–0.99	0.05
Constructional Praxis (z-scores)	0.11	0.57	0.35–0.88	0.01
**Instrumented measures for balance**				
Static sway analysis (DynaPort)				
Sway area (mm^2^)	0.10	1.00	1.00–1.00	0.01
Sway path (mm/s)	0.12	1.09	1.02–1.15	0.01
Medio-lateral displacement, RMS (mm)	0.18	1.25	1.01–1.44	<0.01

Presented are univariate logistic regressions for the significantly discriminating variables (Table 2). Abbreviations: CI: confidence interval; OR: odds ratio: R^2^: Nagelkerkes R^2^; RMS: root mean square.

**Table 4:** Results of the multivariate regression analysis to predict in-hospital falls.

Variable	∆R^2^	OR	95% CI	*p*-Value
Constructional Praxis (z-scores)	0.06	0.62	0.38–1.00	0.05
Medio-lateral displacement, RMS (mm)	0.18	1.23	1.07–1.42	0.01
Total R^2^ = 0.24 (*p* < 0.001)				

Presented are results of the multivariate logistic regression. Abbreviations: CI: confidence interval; OR: odds ratio: R^2^: Nagelkerkes R^2^; RMS: root mean square.
